# American Wine

**Published:** 1863-05

**Authors:** 


American Wine.—We have to acknowledge the receipt of bottles of Williams' Domestic Port Wine, which so far as we are able to judge, is equal to any wine of American manufacture, and it no doubt possesses one advantage at least over most wines sold for *imported,* viz: it is wine, and not a mixture of molasses, whiskey, logwood, alkanet, aloes, etc.

As a medicine, wine was formerly regarded very valuable; but its adulterations, its high price and other causes have lessened its general use.—Possibly the manufacture of pure, choice American wines may lead to a revival of the use of wine more generally as a tonic and stimulant; obviating the more serious obstacles to its general adoption for these purposes.

